# Cannabis Hyperemesis Syndrome: A Psychiatric Approach

**DOI:** 10.7759/cureus.102152

**Published:** 2026-01-23

**Authors:** Bibian K Anibueze, Adebayo Emmanuel

**Affiliations:** 1 Psychiatry, North East London NHS Foundation Trust, London, GBR

**Keywords:** cannabinoid hyperemesis syndrome (chs), cannabis, cannabis use, cyclical abdominal pain, persistent vomiting

## Abstract

Cannabis hyperemesis syndrome (CHS) is a rare complication presenting with recurrent episodes of intractable nausea, vomiting, and abdominal pain that manifests in chronic cannabis use. Here, we report a case of a young male admitted to an inpatient psychiatric ward with drug-induced psychosis, who developed a cyclical vomiting illness on a background of long-term cannabis use. This case, characterised by recurrent emergency department presentations, weight loss, and medical complications, highlights the challenges associated with early recognition and diagnosis of CHS. We discuss the available literature on CHS and proposed management options. We highlight the difficulty of managing substance misuse-related pathologies with the stigma attached, and the importance of collaboration across various disciplines, whilst ensuring a holistic and multidisciplinary approach.

## Introduction

Cannabis hyperemesis syndrome (CHS) was first identified in 2004. It was used to describe a cyclical vomiting illness associated with compulsive bathing in chronic cannabis users, whose symptoms cease on cessation of cannabis use [1].

The clinical course of CHS follows three phases. A prodromal phase, including early morning nausea on some days, precedes a hyperemetic phase involving severe refractory cyclical vomiting, associated with abdominal pain and relieved only by hot baths. The recovery phase follows hyperemesis. Patients usually present to the hospital during the hyperemetic phase and often require parenteral rehydration.

Cannabis has consistently been the most used recreational drug since prevalence estimates began in 1995. As of March 2023, drug misuse statistics in England and Wales highlighted that 8% of adults aged 16-59 and 15% of adults aged 16-24 had used cannabis within the last year [2]. However, cannabis use is not limited to adults; data from a 2021 survey found that 5.6% of pupils aged 11-16 in England used cannabis within the last year [3]. Cannabis use is more prevalent in males compared to females [2].

Cannabis use has increased exponentially in line with the changing times and ever-evolving laws. United Nations data estimate that 219 million people used cannabis worldwide in 2021, compared to 163 million in 2001, with a 21% increase in global cannabis use over the last decade [4]. This is in part due to increasing evidence of the medical benefits of cannabidiol (CBD), the non-intoxicating component of cannabis, in some sub-populations. As a result, cannabis use has been legalised for both medicinal and recreational use in several countries since 2017. In the United Kingdom, cannabis has been available legally for medicinal purposes since November 2018, though its use recreationally remains illegal [5].

Cannabis has not only increased in frequency of use but also in potency. Cannabis used today contains higher concentrations of tetrahydrocannabinol (THC) per gram, as growers select for strains that are highly psychoactive. A systematic review and meta-analysis of delta-9-THC concentrations over time found that the concentration of THC increased by 0.29% each year between 1970 and 2017 [6]. Another study of drug samples seized by the Drug Enforcement Administration in the United States between 1995 and 2014 found that the average CBD concentration had fallen from 0.28% in 1995 to < 0.15% in 2014, whilst the average THC concentration had risen from 4% to 12%. The study also found that the proportion of higher potency samples (7-12% and over 12%) has increased significantly over time [7].

As cannabis use increases, so do the associated medical and psychiatric complications, of which CHS is considered a rare complication. Definitive data on the epidemiology of CHS are lacking; however, a 2018 study evaluating CHS prevalence amongst patients presenting to an emergency department in America found that 32.9% of regular cannabis users, defined as cannabis use for at least 20 days per month, had experienced CHS. The authors extrapolated their findings to estimate that 2.75 million Americans suffer from CHS each year [8]. The veracity of these findings appears to be lacking, and one can question the generalisability of such figures. The authors identified several limitations to the study, including a small sample size, risk of recall bias, and lack of consideration of confounding variables. These all contribute to further doubt regarding these demographic estimates.

Cannabis use is associated with the development of mental illness, particularly the emergence of psychotic disorders, including schizophrenia, and to a lesser extent, the emergence of depression, anxiety disorders, and suicidality [9]*. *As such, cannabis users form a large proportion of patients seen in psychiatry, both in inpatient and outpatient settings. Possible complications of CHS include electrolyte abnormalities, acute kidney injury, and death if untreated. It is therefore crucial that psychiatrists are equipped to recognise CHS early to optimise patient care.

## Case presentation

The patient is a 35-year-old male patient under the community mental health team with diagnoses of 1) mental and behavioural disorder due to the use of cannabinoids and 2) mild learning disability. He had been under mental health services since November 2009, when he presented with anger issues at the age of 17. This was in the context of his father's suicide in October 2007 by jumping off a 12th-storey window. Two years later, he was moved into a 12-storey building by the council. He deteriorated rapidly after this and began using cannabis heavily to cope with the trauma of his father's suicide. Over time, his mental state deteriorated even further. He was treated for low mood and suicidal ideation, became increasingly aggressive, and developed paranoia, nightmares, and auditory hallucinations. Despite referral to drug and alcohol services, the patient continued to use cannabis.

He was treated with risperidone during this period; however, he continued to deteriorate and was detained under the Mental Health Act in July 2015. His presentation at the time was attributed to cannabis-induced psychosis. The patient quickly became non-compliant with prescribed medications after discharge and was detained again under the Mental Health Act in June 2016 and twice more in July 2021 and December 2022. He also accessed crisis services (Home Treatment Team) several times during this period. Between July 2021 and February 2022, the patient was on a community treatment order due to non-compliance with prescribed psychotropics, often in the context of reported side effects. Throughout this, the patient continued to use large amounts of cannabis, quantified as daily use of £20 worth. He did not engage with drug and alcohol service intervention.

During his 23-day admission in December 2022, he began to experience severe nausea and vomiting, requiring multiple medical reviews and transfers to the Emergency Department (ED). The episode started five days after admission, with two bouts of vomiting and some diarrhoea after having a takeaway. On assessment, he did not exhibit signs of an acute abdomen. This presentation was suspected to be gastroenteritis, and conservative management was advised. However, the vomiting persisted, and three days later, blood tests identified Stage 3 acute kidney injury (AKI) secondary to severe dehydration due to recurrent vomiting, along with hyponatraemia, and elevated inflammatory markers. Other investigations, including amylase, bone profile, vitamin B12, folate, and thyroid function tests, were unremarkable, as seen in Table 1. He was transferred to the ED and admitted to a medical ward for nine days, where he was treated with intravenous fluids and antiemetics. Renal function tests improved following intravenous rehydration and were back to normal when repeated six weeks after discharge, as demonstrated in Table 2. 

**Table 1:** Blood results at the onset of illness Table showing lab tests at the point of admission to ED, highlighting the patient was in acute kidney injury (AKI) stage 3, with hyponatraemia and raised inflammatory markers.

Parameter (unit)	Result	Unit	Reference range
Haemoglobin	151	g/L	133-173
White cell count	19.3	10*9/L	3.8-11.0
Neutrophils	15.1	10*9/L	2.0-7.5
Platelets	256	10*9/L	150-400
C-reactive protein	62	mg/L	< 5
Sodium	129	mmol/L	133-146
Potassium	3.6	mmol/L	3.5-5.3
Urea	20.9	mmol/L	2.5-7.8
Creatinine	318	µmol/L	59-104
Estimated glomerular filtration rate (eGFR)	21	mL/min	> 60
Amylase	76	IU/L	28-100
Alanine aminotransferase	13	U/L	< 41
Alkaline phosphatase	79	U/L	30-130
Total bilirubin	8	µmol/L	1-21
Adjusted calcium	2.42	mmol/L	2.20-2.60
Phosphate	2.04	mmol/L	0.8-1.5
Magnesium	1.04	mmol/L	0.7-1.0
Glucose (venous)	5.5	mmol/L	3.5-5.5
Thyroid Stimulating Hormone	0.33	mU/L	0.27-4.2
Vitamin B12	320	ng/L	191-663
Folate	8.8	µg/L	3.9-26.8

**Table 2:** Progression of renal function tests Table highlighting the improvement of renal function following intravenous rehydration.

Parameter (unit)	On admission to ED	At discharge (9 days after)	6 weeks post-discharge	Unit	Reference range
Sodium	129	138	141	mmol/L	133-146
Potassium	3.6	4.8	3.5	mmol/L	3.5-5.3
Urea	20.9	7.1	6	mmol/L	2.5-7.8
Creatinine	318	115	101	µmol/L	59-104
Estimated glomerular filtration rate (eGFR)	21	71	83	mL/min	> 60

A notable observation was made that during admission, the patient's symptoms would improve significantly and subsequently recur on discharge. This was initially ascribed to the discontinuation of antiemetics on discharge, with resultant re-emergence of nausea and vomiting. However, it was later discovered that the critical pattern was cessation of symptoms during periods of abstinence from cannabis, which coincided with hospital admissions, and re-emergence on reinitiation of cannabis use following discharge. This proved to be a key diagnostic feature in later formulations of his illness.

The patient attended the ED 14 times between February 2023 and October 2023, often presenting with AKI and hypokalaemia, secondary to dehydration as a result of intractable vomiting. He often required intravenous rehydration and potassium replacement, after which renal function tests would improve. The patient felt that the nausea and vomiting were side effects of the oral antipsychotic paliperidone. Paliperidone was switched to olanzapine; however, the nausea and vomiting continued despite this. According to the patient, he would vomit up to 20 times on a bad day, and once on a good day during the course of his illness. Nausea and vomiting were associated with recurrent hiccups, severe abdominal pain, fatigue, poor appetite, and weight loss of 20 kg over six months.

**Figure 1:**
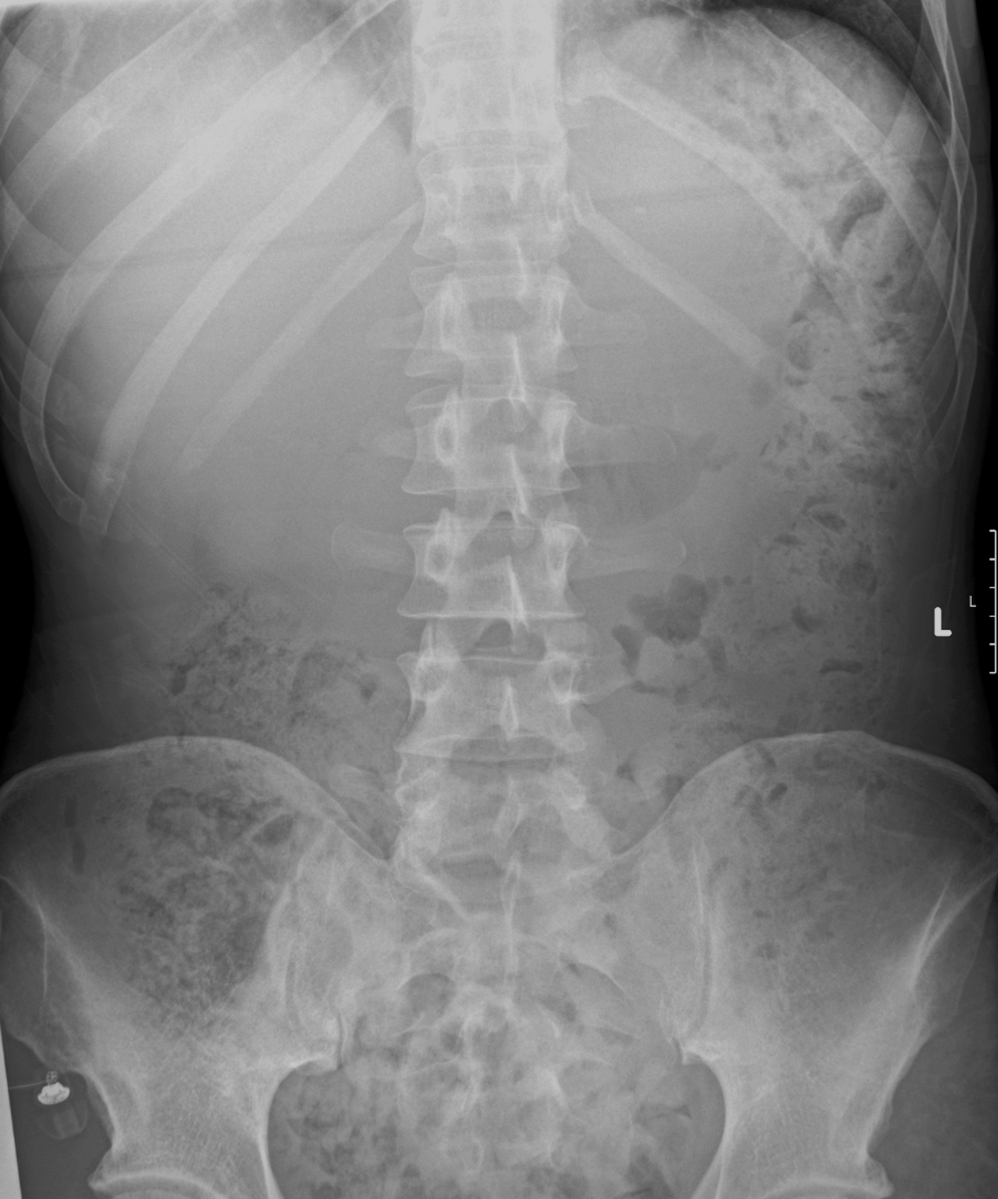
Abdominal X-ray (plain) Findings: There is a 2 mm calcified opacity projected over the left kidney shadow - possible left kidney stone. Moderate faecal loading noted. Opacities in the pelvic cavity are most likely phleboliths.

**Figure 2:**
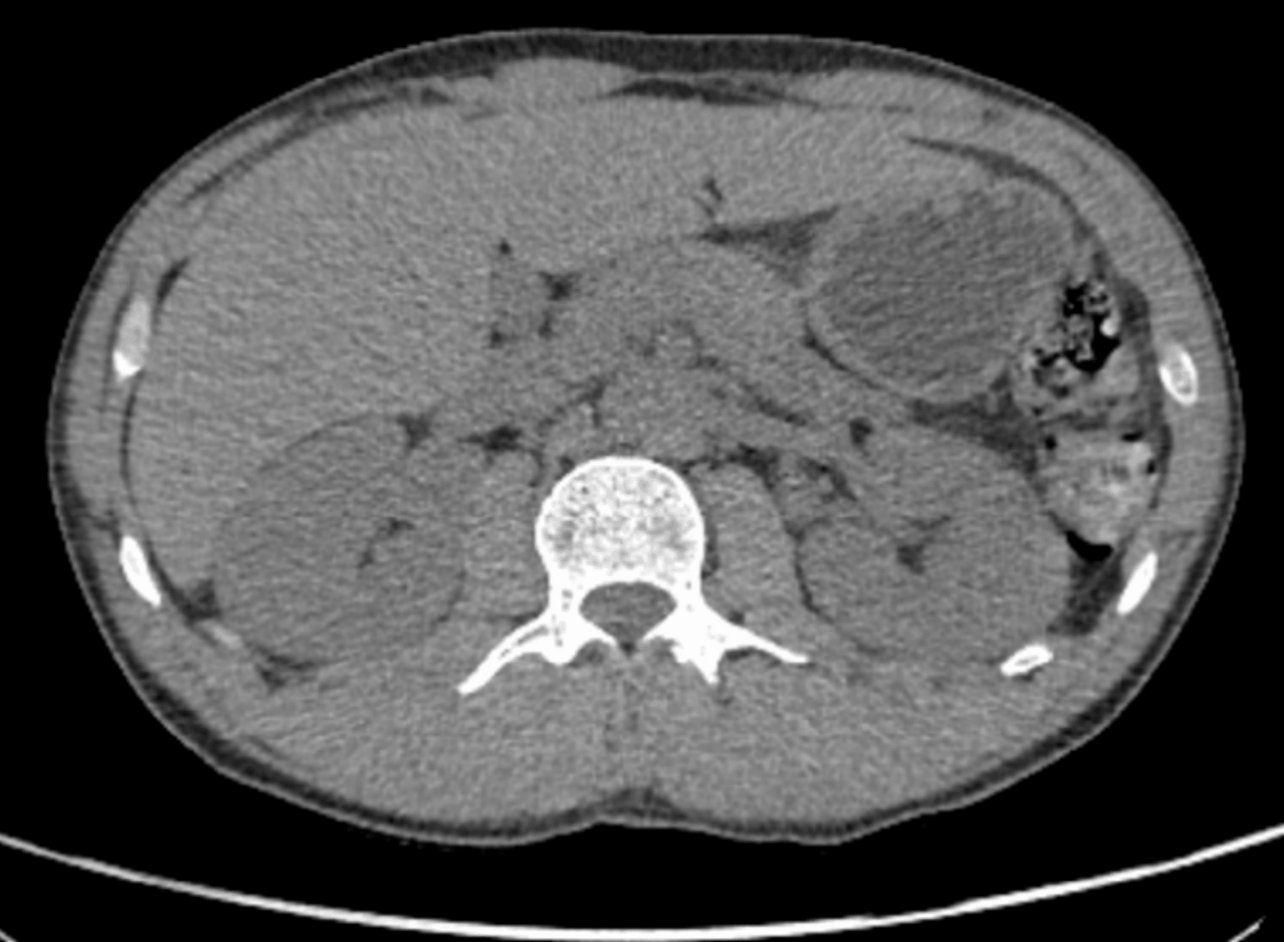
CT abdomen and pelvis (non-contrast) Findings: No hydronephrosis. No bowel obstruction or perforation or free fluid or collection. Bladder catheter is present. Diverticular disease of the large bowel. Normal non-enhanced appearance of the pancreas. No destructive bony lesion. Conclusion: Significant pathology may not be demonstrated on these images. There is no bowel obstruction or perforation and no hydronephrosis.

**Figure 3:**
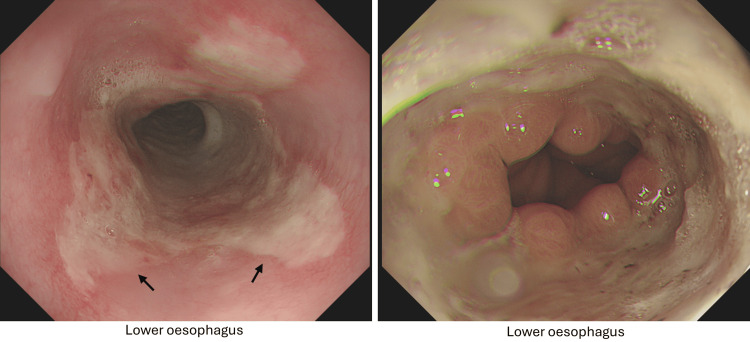
Oesophagogastroduodenoscopy Findings: Oesophagus: Hiatus hernia (sliding of length 2 cm) and oesophagitis with confluent ulceration. Stomach: normal. Duodenum: normal

**Figure 4:**
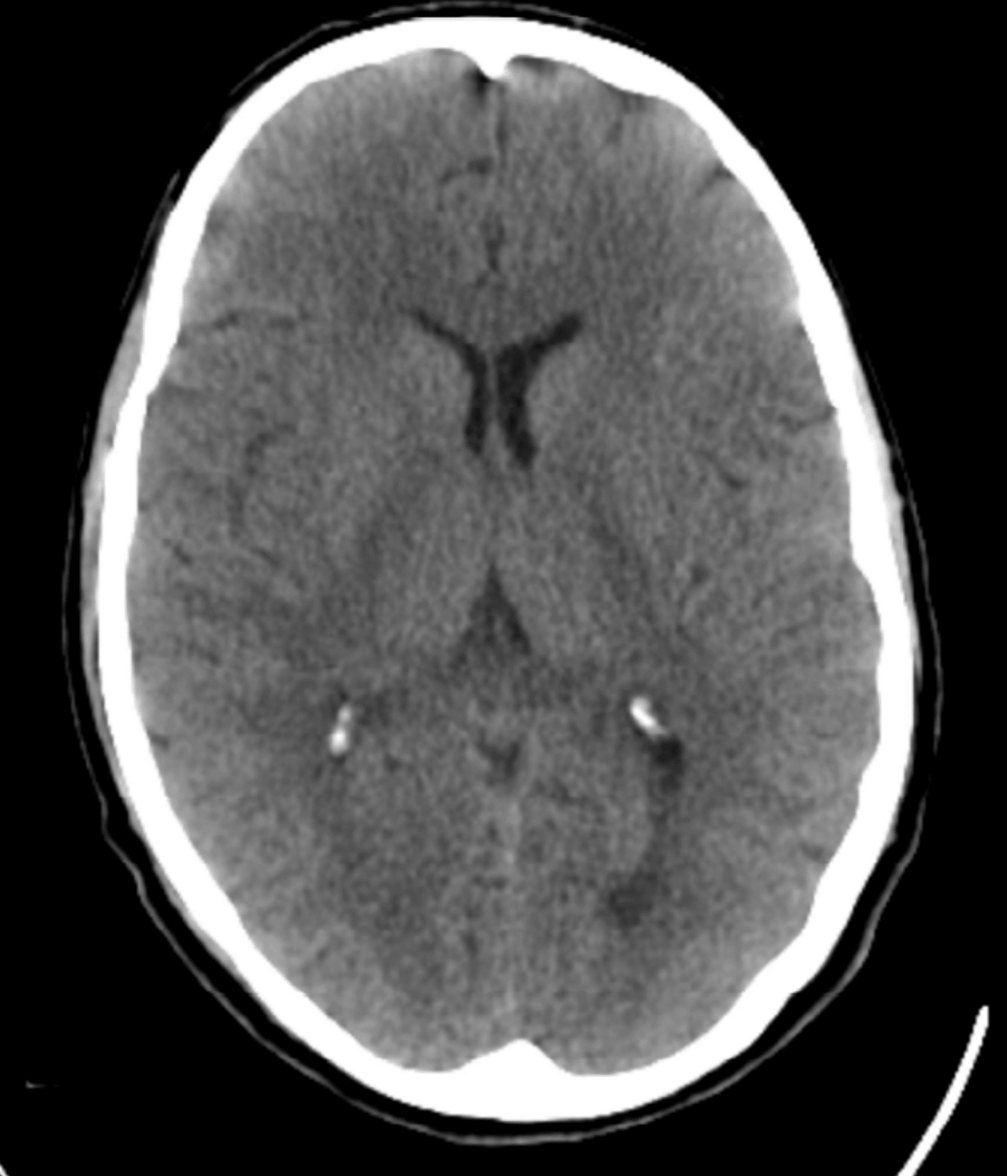
Head CT (non-contrast) Findings: No intracranial bleed or mass. No midline shift. The basal cisterns are patent. No orbital collection or fracture. Minor mucosal thickening in the paranasal sinuses. Mastoid air cells are clear. Conclusion: No intracranial bleed. No intracranial mass.

He was investigated extensively for the aetiology of the vomiting and subsequent recurrent AKIs. Further laboratory testing included autoimmune screen, complement activity, and serum electrophoresis; these results were unremarkable and are demonstrated in Table 3. Abdominal X-ray found an incidental small left kidney stone with moderate faecal loading, as seen in Figure 1. Computed tomography (CT) of the abdomen and pelvis, illustrated in Figure 2, highlighted diverticular disease, though this did not account for the persistent vomiting presentation. An oesophago-gastro-duodenoscopy (OGD) highlighted oesophagitis with confluent ulceration and an incidental finding of a small sliding hiatus hernia, as shown in Figure 3. These were not deemed clinically significant causative factors for the persistent vomiting and were instead considered likely secondary to the intractable vomiting. Following OGD, the patient was started on high-dose proton pump inhibitor therapy, specifically omeprazole 40 mg twice daily. However, this did not contribute to a significant improvement in symptoms, supporting the conclusion that oesophagitis was not the causative pathology. Gastric emptying studies were not conducted during his work-up. Finally, a non-contrast head CT scan did not identify any significant pathology, as demonstrated in Figure 4. 

**Table 3:** Autoimmune screen, complement activity, and serum electrophoresis Connective tissue disease antibodies include anti-double-stranded deoxyribonucleic acid antibodies (dsDNA), anti-smith antibodies (Sm), anti-ribosomal P antibodies, anti-proliferating cell nuclear antigen antibodies (PCNA), anti-U1 small nuclear ribonucleoprotein antibodies (U1-snRNP), anti-ro antibodies (Ro), anti-la antibodies (La), anti-topoisomerase I antibodies (Scl-70), Anti-centromere antibodies, anti-fibrillarin antibodies, anti-ribonucleic acid polymerase III antibodies (RNA pol III), anti-histidyl transfer ribonucleic acid synthetase antibodies (Jo-1), anti-chromodomain-helicase-deoxyribonucleic acid-binding protein 4 antibodies (Mi-2), anti-polymyositis/scleroderma antibodies (PM/Scl).

Parameter	Result	Unit	Reference range
Anti-nuclear antibody	Negative	-	Negative
Anti-gastric parietal cell antibody	Negative	-	Negative
Anti-mitochondrial antibody	Negative	-	Negative
Anti-smooth muscle antibodies	Negative	-	Negative
Anti-liver-kidney microsomal antibody	Negative	-	Negative
Connective tissue disease antibodies	0.2	U/mL	<1
Cytoplasmic anti-neutrophil cytoplasmic antibody	0.3	U/mL	<2.0
Perinuclear anti-neutrophil cytoplasmic antibody	0.3	U/mL	<3.5
Anti-glomerular basement membrane antibodies	< 0.8	U/mL	<7
Complement component C3	1.30	g/L	0.90-1.80
Complement component C4	0.31	g/L	0.10-0.40
Immunoglobulin G (IgG)	9.02	g/L	5.5-16.5
Immunoglobulin A (IgA)	2.33	g/L	0.80-4.00
Immunoglobulin M (IgM)	0.51	g/L	0.50-1.90

During a routine community mental health outpatient appointment, it became apparent that abstinence from cannabis use led to a complete resolution of symptoms, compared to whenever he reinitiated cannabis use. The patient had not used cannabis in five months at the time of the review and had remained asymptomatic during the period of abstinence. It was also noted that the patient was having at least five hot showers or baths daily when symptomatic, as he found these soothed his symptoms. Hence, the patient was diagnosed with cannabis hyperemesis syndrome (CHS) in September 2023, nine months after his symptoms emerged.

The patient was prescribed capsaicin cream, which he found helpful in conjunction with frequent baths to ease his symptoms. He was advised to abstain from cannabis use permanently; however, he continued to use cannabis, and the symptoms persisted. It is important to note that patient reports during outpatient appointments suggested he was indeed abstinent; however, collateral from family refuted this and confirmed the patient was using cannabis daily. As a result, the symptoms continue to affect his quality of life, with emergent suicidal ideation. A comprehensive crisis plan has been developed in co-production with the patient to manage his deteriorating mental health. This includes admission under the psychiatric home treatment team for community-based intensive support when required, access to 24-hour crisis hotlines, and regular oversight by a psychiatric community care coordinator to monitor and mitigate mental health risks.

The patient continues to frequently attend the ED due to intractable vomiting and abdominal pain at the time of this paper being written. The London Ambulance Service is aware of his diagnosis, given frequent attendances and conveyances to the ED, and local services are currently working on a joint care plan involving all the relevant organisations to minimise attendances at the ED. He is prescribed meal replacement therapy in view of significant weight loss and has regular general practice and psychiatry reviews.

## Discussion

Prompt recognition of CHS is often difficult, owing in part to a lack of awareness about the condition, as well as the plethora of differential diagnoses in patients presenting with vomiting and abdominal pain. This is especially challenging in psychiatric populations where there is a complex interplay between trauma, substance misuse, and psychiatric presentations. In this case, cannabis use began as a maladaptive coping mechanism for trauma. In addition, the patient's symptoms were initially attributed to possible psychotropic-induced side effects. This highlights a pertinent diagnostic dilemma in which psychiatrists must differentiate between trauma-related functional presentations, medication-induced adverse effects, and organic complications of substance use such as CHS. In psychiatric settings, CHS is often misdiagnosed as psychogenic vomiting after initial investigations come back unremarkable without medical causes being evident.

To complicate matters, cannabis has antiemetic properties for which it has been utilised medically in oncology settings to manage chemotherapy-induced nausea and vomiting [10]. Additionally, there have been case reports of cannabis being utilised by patients with hyperemesis gravidarum with good effect [11], though no trials have been completed evaluating this practice. Cannabis’ role as an antiemetic poses a paradoxical conundrum of how cannabis can both induce and limit vomiting.

The pathophysiology of CHS remains unknown. Many theories have been proposed, one of which is Delta-9-THC toxicity from chronic cannabis use leading to increased activity at the cannabinoid receptor 1 (CB1) [12]. This theory is supported by the increased concentrations of THC in cannabis today, compared to the cannabis used in the research, thus corroborating cannabis’ antiemetic properties. Nevertheless, it remains unclear why only a small proportion of CHS is seen in clinical settings, compared to the large proportions of the population that use cannabis.

CHS presents similarly to cyclical vomiting syndrome (CVS), a functional disorder that presents with cyclical vomiting. This poses a diagnostic challenge in patients presenting with cyclical vomiting on a background of cannabis use, as it becomes unclear whether they have CHS due to cannabis use or CVS managed with cannabis use. Nevertheless, resolution of symptoms upon cessation of cannabis use remains a distinguishing diagnostic feature.

One of the key difficulties in the diagnosis of CHS is the lack of defined diagnostic criteria. A large systematic review identified the four main characteristics associated with CHS with the highest sensitivity: Severe cyclical vomiting often associated with abdominal pain, at least weekly cannabis use for over one year, compulsive hot baths or showers with temporary symptom relief, and complete symptom resolution on cessation of cannabis use [12].

The Rome IV Criteria were defined in 2016 for functional gastrointestinal disorders and are currently the accepted diagnostic checklist for cannabis hyperemesis syndrome. The checklist includes a three-part diagnostic checklist, of which all three must be present. The checklist includes 1) cyclical vomiting similar to CVS, 2) symptom onset preceded by chronic cannabis use, and 3) symptom resolution on cessation of cannabis. Diagnosis of CHS requires a minimum of six months' history of illness, with at least three months of criteria fulfilment [13].

Despite the development of criteria, CHS remains under-recognised. This was evident in the case presented, with a nine-month period from symptom onset to diagnosis. A unique challenge in the diagnosis or recognition of CHS lies in the fact that the diagnosis relies completely on an accurate and thorough drug and alcohol history. Patients who misuse illicit substances are often unwilling to disclose this, and when they do, they often underreport the quantity of substance misuse. Perceived stigma and concerns over the potential criminality involved are two reasons this may be. Furthermore, some clinicians may not feel adept at taking an in-depth substance misuse history, particularly in the context of vomiting as the presenting complaint. Adequate communication skills, including signposting and reassuring confidentiality, would be useful here. This is particularly pertinent in psychiatric settings in which patients may be on community orders precluding substance misuse. In the case presented, cannabis use was evident from the onset of symptoms.

During the nine months between symptom onset and diagnosis, the patient attended the ED several times and continues to do so, incurring further costs to the National Health Service (NHS). The patient had several investigations, including an OGD, one abdominal X-ray, three abdominal CT scans, and four abdominal ultrasounds over a seven-month period. This delay in diagnosis is mirrored in the literature, with an average of 4.1 years between symptom onset and diagnosis highlighted in one study [12]. Another study highlighted the economic burden of CHS, with an average of 17.9 ED visits prior to diagnosis, 4.9 CT scans, and 2.4 ultrasounds. The average cost incurred per patient in that study was roughly $76,000 [14].

There are currently no economic studies quantifying the costs of CHS to the NHS in the United Kingdom (UK). This represents an important gap in the literature, particularly in view of the legal availability of cannabis for medicinal purposes within the UK. Further research is therefore warranted to establish the economic burden of CHS locally. Nevertheless, based on the available studies from the United States, it is evident that the economic burden of CHS is one of a great magnitude, further emphasising the utility of early recognition, diagnosis, and management of CHS.

In psychiatric inpatient settings, there is seldom adequate equipment to manage physical health emergencies, some of which may present as complications of CHS. It is therefore inevitable that patients will need to be transferred to the ED for medical optimisation. Notwithstanding, psychiatrists have a key role to play in early recognition. Urine drug screens are routinely done on admission to inpatient units, giving the psychiatrist knowledge of patients’ cannabis misuse status, which aids in diagnosis. Education and training for psychiatrists on the presentation and diagnostic criteria of CHS will prove beneficial in enhancing patient care.

It is also worth noting the psychiatric implications of physical illness. CHS can result in electrolyte imbalance, some of which can worsen mental illness presentations. Moreover, it is well known that chronic physical health conditions are associated with a deterioration in one's mental state, as evidenced by worsening suicidal ideation in the patient following the onset of CHS symptoms. Psychiatrists' duty of care to patients extends to the entirety of the patient’s health, and adequate management of CHS will, in turn, benefit their mental health.

Management of CHS proves challenging as symptoms often do not respond to conventional antiemetics. The mainstay of CHS management is the cessation of cannabis use. Due to the half-life of THC, it may take a few weeks of abstinence from cannabis for symptoms to resolve completely [15]. Even so, cessation of cannabis use is the most effective management strategy [16].

Consideration should be given to the emergence of cannabinoid withdrawal syndrome, characterised by poor appetite, anxiety, irritability, low mood, and sleep disruption. This is particularly useful in the consideration of barriers to patients' abstinence from cannabis. Patients should be referred to the appropriate drug and alcohol services for support with substance use. Management of co-morbid psychiatric disorders, including anxiety, depression, and trauma-related presentations, is also important to consider [17].

Studies have found tricyclic antidepressants, particularly amitriptyline, to be effective prophylaxis against cyclical vomiting in CHS. It is particularly useful in mitigating symptoms associated with cannabis withdrawal during the discontinuation period. Slow dose titration beginning at 10 mg daily is advised to minimise anticholinergic side effects and overall anticholinergic burden, with effective doses usually between 50 and 200 mg per day. Amitriptyline is typically continued and can be weaned off once the patient has been in remission for 6-12 months [17].

Other treatment options identified in the literature, which provide symptomatic relief in CHS, include hot water hydrotherapy, benzodiazepines, capsaicin cream, haloperidol, droperidol, propranolol, and aprepitant [18]. Topical capsaicin cream, known to act on the peripheral tissue receptor, transient receptor potential vanilloid type 1 (TRPV1), has proven beneficial in the management of abdominal pain in CHS compared with conventional analgesics [19]. Hot water exposure above 41 degrees Celsius has also been linked to the TRPV1 receptor [19]. The TRPV1 receptor has been shown to inhibit the release of substance P associated with inflammation and pain, as well as interact with the endocannabinoid system, believed to be the primary driver of CHS [18-20]. Proton pump inhibitor use has also been proposed in CHS to minimise the risk of oesophageal or gastric lesions secondary to hyperemesis [19].

In the case reported, the patient benefited from capsaicin cream and self-administered hot water hydrotherapy via frequent hot baths. In some cases, patients have sustained severe burns from incredibly hot baths [1,19]; a complication that is important for healthcare professionals to be aware of and counsel patients on. This is equally pertinent in consideration of patients' admission to hospitals, as hospitals or centres with bath or shower facilities can be prioritised in hospital flow discussions.

Psychological support, including motivational interviewing, can be a useful tool in limiting one’s use of cannabis. A systematic review assessing the effectiveness of motivational interviewing on cannabis use highlighted a reduction in the frequency and quantity of cannabis use among adults who underwent motivational interviewing [21]. Psychoeducation is critical to this, employing a fully bio-psycho-social model of healthcare to empower patients to abstain from cannabis use.

Managing patients' underlying trauma is also essential, given the established relationship between traumatic experiences and substance use disorders. Research has shown that exposure to trauma, particularly during childhood, increases the risk of substance use and the severity of subsequent substance use disorders. It is therefore paramount that trauma-informed approaches are utilised in the management of substance use disorders [22,23]. A study comparing integrated treatment approaches for trauma presentations and substance use found that integrated treatment was more effective in reducing long-term substance use compared with trauma-focused cognitive behavioural therapy alone [24]. This emphasises the importance of in-depth assessment of trauma symptoms and their concurrent management within substance use treatment strategies.

## Conclusions

CHS is an important and under-recognised condition that is particularly relevant in psychiatric populations where cannabis use is prevalent. It has a significant impact on health and associated high costs of service utilisation, as demonstrated in international studies. With increasing cannabis use and legalisation across many countries, it is imperative that psychiatrists possess the ability to recognise and manage this debilitating condition promptly. In our case, the diagnosis was supported by the triad of findings, including extensive investigations excluding significant organic pathology, complete symptom resolution during five months of abstinence, and consistent symptom recurrence upon cannabis re-use. Among these, resolution of emetic symptoms on cessation of cannabis use remains the key distinguishing diagnostic feature of CHS. Abstinence remains the primary treatment measure, a feat that proves increasingly challenging in today's climate. Despite enrolment in treatment programmes, relapse is common. Thus, a multidisciplinary approach is fundamental to achieving long-term treatment efficacy and relapse prevention, with psychoeducation of patients and their families, as well as trauma-informed practice, at the core of treatment strategies.
